# Maximum Likelihood Estimation of the Negative Binomial Dispersion Parameter for Highly Overdispersed Data, with Applications to Infectious Diseases

**DOI:** 10.1371/journal.pone.0000180

**Published:** 2007-02-14

**Authors:** James O. Lloyd-Smith

**Affiliations:** Center for Infectious Disease Dynamics, Mueller Lab, Pennsylvania State University, University Park, Pennsylvania, United States of America; University of Sheffield, United Kingdom

## Abstract

**Background:**

The negative binomial distribution is used commonly throughout biology as a model for overdispersed count data, with attention focused on the negative binomial dispersion parameter, *k*. A substantial literature exists on the estimation of *k*, but most attention has focused on datasets that are not highly overdispersed (i.e., those with *k*≥1), and the accuracy of confidence intervals estimated for *k* is typically not explored.

**Methodology:**

This article presents a simulation study exploring the bias, precision, and confidence interval coverage of maximum-likelihood estimates of *k* from highly overdispersed distributions. In addition to exploring small-sample bias on negative binomial estimates, the study addresses estimation from datasets influenced by two types of event under-counting, and from disease transmission data subject to selection bias for successful outbreaks.

**Conclusions:**

Results show that maximum likelihood estimates of *k* can be biased upward by small sample size or under-reporting of zero-class events, but are not biased downward by any of the factors considered. Confidence intervals estimated from the asymptotic sampling variance tend to exhibit coverage below the nominal level, with overestimates of *k* comprising the great majority of coverage errors. Estimation from outbreak datasets does not increase the bias of *k* estimates, but can add significant upward bias to estimates of the mean. Because *k* varies inversely with the degree of overdispersion, these findings show that overestimation of the degree of overdispersion is very rare for these datasets.

## Introduction

The negative binomial (NB) distribution has broad applications as a model for count data, particularly for data exhibiting overdispersion (i.e. with sample variance exceeding the mean). In the biological literature, classical uses of the NB distribution include analysis of parasite loads, species occurrence, parasitoid attacks, abundance samples and spatial clustering of populations [1]–[7]. The range of applications of the NB distribution was extended recently to include the epidemiology of directly-transmitted infections, as the NB distribution was shown to be a suitable model for the ‘offspring distribution’ for a number of disease transmission datasets [8]. The offspring distribution, a concept arising in the theory of branching processes [9], is the probability distribution for the number of individuals (termed ‘secondary cases’) infected directly by each infectious individual in a disease outbreak. Estimation of NB parameters for empirical offspring distributions revealed a high degree of overdispersion—particularly for severe acute respiratory syndrome (SARS), measles, and smallpox—signalling an unexpectedly large influence of individual variation and ‘superspreading’ on the dynamics of disease emergence [8]. However, the authors emphasized the challenges inherent in estimating NB parameters and the confidence intervals (CIs) associated with those estimates, and noted that previous work on NB parameter estimation had not explored the parameter ranges of interest for epidemiological studies. A particular concern is whether the results were influenced by small sample size in the datasets analyzed, or biases peculiar to disease transmission data. This study uses simulated data to assess the bias and precision of NB parameter estimates and the coverage accuracy of CIs for highly overdispersed datasets, addressing the challenges of small datasets as well as potential biases arising in the data collection process.

The popularity of the NB distribution is due largely to its ability to model count data with varying degrees of overdispersion. The distribution is commonly expressed in terms of the mean *m* and dispersion parameter *k* such that the probability of observing a non-negative integer *x* is
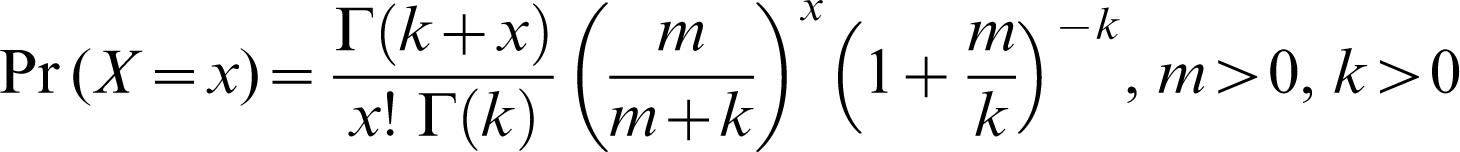
1The variance of the NB distribution is *m* (1+*m*/*k*), and hence decreasing values of *k* correspond to increasing levels of dispersion. The Poisson distribution is obtained as *k*→∞, and the logarithmic series distribution is obtained as *k*→0 [1], [10]. When *k* = 1, the NB distribution reduces to the geometric distribution. Note that recent work in the statistical literature uses the quantity α = 1/*k* due to its preferable properties for inference (discussed below), but studies applying the NB distribution in ecology and epidemiology are overwhelmingly posed in terms of *k*. Accordingly, all calculations in this study were conducted using α, but all results and discussion are posed in terms of *k*. (Confusingly, the term ‘dispersion parameter’ can refer to either *k* or α; other terms for *k* include ‘shape parameter’ and ‘clustering coefficient’.)

The dispersion parameter *k* is commonly used as an inverse measure of aggregation in biological count data [1]–[5], [8], [11], [12], yet its estimation from finite datasets is a recognized challenge. Many simulation studies have examined the efficacy of different estimators of NB parameters for finite datasets [11], [13]–[16], [17; also see review in 14], but owing to precedent most of these have focused on *k*≥1 and hence do not apply to highly overdispersed data. One biologically motivated study did explore values of *k*<1 [16], but it did not test the maximum-likelihood (ML) methods of estimation that have become standard owing to their asymptotic efficiency and low bias [12], [13], [17]. The small-sample accuracy of ML estimates of *k* has not been tested for NB distributions with moderate to high degrees of overdispersion. Moreover, little attention has been paid to the accuracy of CIs of such NB parameter estimates. The first aim of this study, therefore, is to investigate the bias, precision and CI coverage accuracy of ML estimates of *k* for small samples. The investigation focuses on datasets with *k*<1, to address the gap in existing studies, but results for *k*≥1 are included to establish continuity with earlier work.

The second aim is to investigate how estimates of *k* are affected by potential biases of the data collection process, in particular systematic under-counting of events and the selection bias inherent in disease outbreak data. The disease transmission datasets analyzed by Lloyd-Smith et al. [8] fell into two broad categories, surveillance and outbreak datasets, each of which presents challenges due to the processes by which data are generated and collected.

Surveillance datasets combine information about many separate introductions of a disease into a population of hosts. Empirical offspring distributions can be constructed by counting the number of secondary cases infected by the first infectious individual in each outbreak, but ignoring all subsequent generations of transmission (which often are not reported in detail, or may be influenced by outbreak control measures). The resulting datasets are analogous to many other datasets in biology, compiling many independent records of unrelated events. Datasets of this type can be affected by two broad classes of under-counting error. First, data points may be underestimated, due to the possibility that some of the secondary cases will be overlooked, misdiagnosed, or not traced to the individual that infected them. Second, individuals who do not transmit the disease may be more likely to be missed by surveillance programs, because they do not initiate a cluster of cases and thus are less likely to attract the attention of health authorities. Therefore instances of a particular value (i.e. *x* = 0, for no secondary cases) may be systematically under-counted in the surveillance samples. These two classes of under-counting error are common to many types of biological data [e.g. 18], [19], [20].

Outbreak datasets, comprising the second category of disease transmission data, are more unique to epidemiology and disease ecology. Offspring distributions drawn from outbreak data include the number of secondary cases caused by many individuals within a single disease outbreak. These datasets arise when several generations of epidemic spread (typically early in an outbreak, before control measures are imposed) are fully reconstructed by contact tracing, so the number of secondary cases caused by each infectious case can be determined. Lloyd-Smith et al. [8] showed that when the degree of infectiousness is highly overdispersed (e.g. when the offspring distribution is NB with *k*<1), many outbreaks will die out stochastically in their first few generations of spread. In such situations, the outbreaks that survive tend to be those where a highly infectious individual (i.e. an individual whose number of secondary cases is drawn from the right-hand tail of the offspring distribution) appears in the early generations [8]. Because outbreak datasets necessarily are drawn from successful outbreaks, there is the possibility of selection bias for an increased proportion of exceptionally infectious individuals, or ‘superspreaders’ [21]. Intuitively, this risk appears to be particularly acute for offspring distributions with lower mean values, for which the epidemic's growth is more dependent on chance. (Note that the mean of the offspring distribution corresponds to the basic reproduction number *R*_0_ of the disease [8], [22]).

## Methods

### 2.1 Generating simulated data sets

Four types of simulated datasets were examined. In all cases, the datasets comprised *n* values, *x_i_* (*i* = 1, 2, …, *n*), generated as described below. In the epidemiological context that motivated this study, these values *x_i_* correspond to the numbers of secondary cases that were infected by *n* different infectious individuals, but similar data could arise from many other processes. All simulations were conducted using Matlab v6.1 (MathWorks, Cambridge MA).

#### 2.1.1 Negative binomial data

Because the NB random number generator in Matlab v6.1 (nbinrnd) does not allow non-integer values of *k*, NB random variates were simulated using the fact that the NB distribution can be derived as a Poisson distribution with gamma-distributed intensity, i.e. a Poisson-gamma mixture [23], [24]. First, *n* values *g_i_* were drawn from a gamma distribution with mean *m* and dispersion parameter *k*. Second, each of these values was used as the intensity parameter for a Poisson random variate to yield a NB-distributed value *x_i_*, i.e. *x_i_* = Poisson(*g_i_*). Random variates were generated using the Matlab functions gamrnd and poissrnd.

#### 2.1.2 Negative binomial data with uniform under-counting

To simulate surveillance datasets with uniform under-counting of data, it was assumed that each secondary case can be missed by surveillance with a fixed probability *p_u_*. Raw data were drawn from a NB distribution with parameters *m* and *k*, as described in section 2.1.1 above. Each value *x_i_* was then decreased by an amount *d_i_*∼binomial(*x_i_*, *p_u_*), generated using the Matlab function binornd, to represent under-counting.

#### 2.1.3 Negative binomial data with under-reporting of zeroes

To simulate the possible under-reporting of individuals who cause no secondary infections, it was assumed that all individuals who caused *x_i_* = 0 cases can be overlooked with some fixed probability *p_z_*, while all other individuals have their full case-count recorded. NB samples were generated as in section 2.1.1, then any value *x_i_* = 0 was deleted with probability *p_z_* and replaced by another NB random variate. If the new value was also 0, then it was again replaced with probability *p_z_*. This process was repeated until a sample of *n* values was generated, in which each remaining value *x_i_* = 0 had avoided replacement exactly once.

#### 2.1.4 Outbreak data

To generate outbreak datasets, stochastic disease outbreaks were simulated as discrete-time branching processes with NB offspring distributions, using the method described by Lloyd-Smith et al. [8]. Each outbreak was assumed to begin with a single infected individual, who transmits the disease to *x*_1_ other individuals, where *x*_1_ is drawn from a NB distribution with parameters *m* and *k*. Each of these second-generation cases infects *x_i_* other individuals, where the *x_i_* are independent and identically distributed draws from the same NB offspring distribution; the number of cases in the third generation is then 
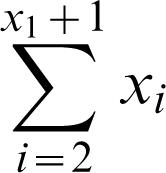
. This process was repeated until the cumulative number of cases exceeded *n*, and the *x_i_* values corresponding to the first *n* infectious cases were used as the simulated outbreak dataset. To mirror the selection bias in using real outbreak datasets of a given size, outbreaks were simulated repeatedly until the cumulative number of cases was *n* or higher. Outbreaks that died out with fewer than *n* total cases were not used. Large outbreaks were less likely when *m*<1, particularly for *k*>1 where extremely infectious individuals (who cause large superspreading events) were very rare. No results were reported for parameter sets for which fewer than 1 in 10^5^ simulated outbreaks had *n* cases or more. Otherwise, simulations were repeated until the desired number of datasets was obtained.

### 2.2 Estimation of dispersion parameter and confidence interval

For each of the above classes of simulated data, 10,000 simulated datasets were generated for each combination of the mean *m* = {0.5, 1.0, 3.0}, the dispersion parameter *k* = {0.1, 0.3, 0.7, 1.0, 3.0, 10.0}, and the sample size *n* = {10, 30, 100, 300}, in a full factorial design. Datasets with no non-zero values of *x_i_* were rejected, as *k* cannot be estimated from all-zero data. For each simulated dataset, the ML estimate *k̂* was determined as described below. The 90% CI was calculated, and it was recorded whether the true value *k* fell within the CI, above its upper bound (termed a CI underestimate), or below its lower bound (a CI overestimate). The 90% CI was studied instead of the 95% interval because the more extreme values of *k* are most difficult to estimate accurately, and to match results presented in Lloyd-Smith et al. [8].

An extensive statistical literature exists on ML estimation of NB parameters [1], [10], [11], [13], [15], [17]. This work shows that it is better to make inferences about *k* indirectly via its reciprocal α = 1/*k*, for two reasons. First, use of the reciprocal avoids discontinuities for homogeneous datasets, because increasing homogeneity yields α→0 instead of *k*→∞. Indeed, there is a continuous transition to values α<0 corresponding to underdispersion (when sample variance is less than the mean), for which direct estimation of *k* is problematic [14], [25]. Second, the sampling distribution for α tends to be more symmetric than that for *k* [13] (an example using outbreak data is shown in Fig. SI-1 of Lloyd-Smith et al. [8]).

In this study ML estimation was conducted for the parameter α, but results are reported in terms of *k̂* = 1/α̂ because *k* is more familiar to epidemiologists and ecologists. Estimates of α̂ were restricted to positive values, because the allowed range for *k* was (0,∞). Underdispersed datasets were assigned the minimum value of α̂, corresponding to *k*→∞. This approximation is reasonable because the study focuses on highly overdispersed NB distributions (with *k*<1); estimation of α̂ for underdispersed data is discussed in-depth elsewhere [14], [15], [17], [25]. The ML estimate of *m* is the sample mean, *x* ¯ [10]. The ML estimate of α was determined by unidimensional numerical maximization of the log-likelihood function [15], conducted using the fminbnd function of Matlab 6.1 over the interval (0.001,1000). The termination tolerance was set sufficiently small that negligible accuracy was lost in inverting the estimates, and direct ML estimates of *k* (obtained by maximizing the log-likelihood function derived from equation (1)) matched *k̂* = 1/α̂ to beyond the fourth decimal place. Reported estimates of *k̂* thus are drawn from the range (0.001,1000), which is much broader than the range of *k* commonly estimated from epidemiological data (e.g. the range of *k̂* was [0.032,5.1] in 11 uncontrolled outbreak datasets [8], or [0.038,6.014] in 49 macroparasite burden datasets [4]). NB distributions with *k* = 1000 and *k*→∞ (the Poisson distribution) are indistinguishable in practice.

Confidence intervals for *k̂* were estimated from the asymptotic variance of the sampling distribution, given by the inverse of the information matrix [24]. For 11 outbreak datasets, intervals estimated in this way were very similar to those estimated using bias-corrected bootstrap methods (both parametric and non-parametric) and asymptotic variance for the zero-class estimator of *k* [8]. For ML estimates of *k̂* or α̂, the asymptotic sampling variances (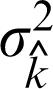 or 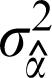) cannot be expressed in closed form but are easily calculated numerically [10], [17]. These variances are related by 
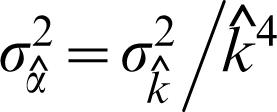
 [13]. In this study 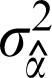 was calculated for each simulated dataset, and the 90% CI for α̂ was estimated as [α̂−*z*_0.95_σ_α̂_, α̂+*z*_0.95_σ_α̂_], where *z*_0.95_ is the 95^th^ percentile of the standard normal distribution [24]. The CI for *k̂* was generated by inverting and reversing the endpoints of the interval for α̂. When α̂−*z*_0.95_σ_α̂_<0, the upper bound of the interval for *k̂* was assumed to be *k*→∞.

## Results

### 3.1 Negative binomial data

**Figure 1:**
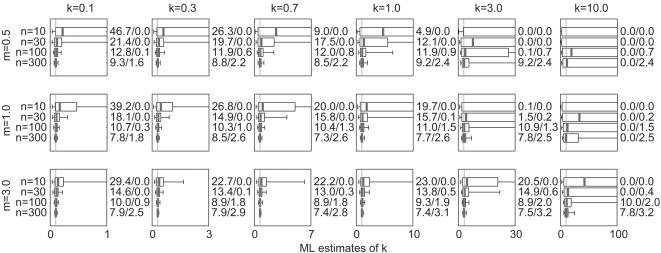
Estimated values of *k̂* and confidence interval coverage for NB datasets. 10,000 datasets were simulated as described in Section 2.1.1 of the text, using mean *m*, dispersion parameter *k*, and sample size *n* as shown. Boxes show the median and interquartile range (IQR) of 10,000 resulting ML estimates of *k̂*, and whiskers show the 5^th^ and 95^th^ percentile values. Numbers to the right of each subplot show the percentage of simulations for which the true value of *k* was outside (below (CI overestimate)/above (CI underestimate) for the numbers *y*/*z*, respectively) the 90% confidence interval estimated for *k̂* The vertical line in each subplot shows the true value of *k*. To facilitate comparison among parameter sets, the horizontal axis of all subplots is scaled from 0 to 10 times the true value of *k*.

The results for unaltered NB datasets are shown in Figure 1. Boxplots show the median, interquartile range (IQR) and [5^th^, 95^th^] percentile interval of 10,000 ML estimates *k̂* for each parameter set, while vertical lines show the true value of *k*. In general, the estimates are biased upward (i.e. favoring values *k̂*>*k*) but converge on the true value *k* as sample size *n* increases. For a given *n*, estimation tends to be less biased (the median value of *k̂* is closer to *k*) and more precise (the IQRs of *k̂* are smaller) for larger values of *m* and smaller values of *k*.

Numbers to the right of each subplot in Figure 1 show the coverage accuracy of the CIs estimated for *k̂*. The two numbers *y*/*z* show, respectively, the percentage of simulations for which the true value of *k* fell below and above the estimated CI. For the 90% intervals estimated here, perfect coverage would yield values 5.0/5.0. For almost all parameter sets the proportion of CI overestimates (when the lower bound of the CI exceeds the true *k*) is greater than 5%, sometimes substantially so. This pattern is broken only for small *n* and large *k*. For all parameter sets the proportion of CI underestimates (when the upper bound of the CI is below the true *k*) is less than 5%. When the proportion of CI overestimates is very high (>10%, say), CI underestimates tend to be almost non-existent. The true coverage of the estimated 90% CIs (calculated as (100−*y*−*z*)%) is generally less than 90%, although it often approaches this value for *n* = 300. Again, there is an exception for small *n* and large *k*, when realized coverage exceeds 90% and reaches 100% in some instances (when the CI is extremely broad).

### 3.2 Negative binomial data with uniform under-counting

**Figure 2:**
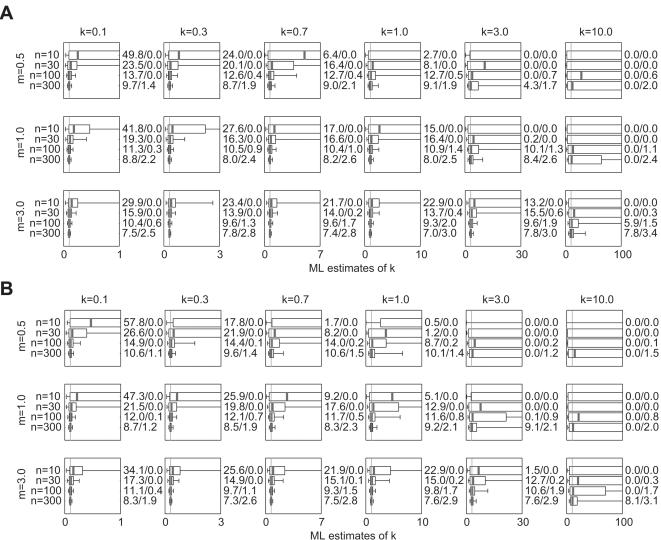
Estimated values of *k̂* and confidence interval coverage for NB datasets with uniform under-counting of secondary cases. The probability with which any secondary case was missed by surveillance was (a) *p_u_* = 0.2 and (b) *p_u_* = 0.5. 10,000 datasets were simulated as described in Section 2.1.2 of the text, for parameters *m*, *k*, and *n* as shown. Plotting details are described in Figure 1.

The results for NB surveillance datasets subject to uniform under-counting are shown in Figure 2. Results are shown for two values of the probability *p_u_* that any given secondary case is missed by surveillance. When *p_u_* = 0.2 (Fig. 2a), estimates of *k̂* from these datasets differed only slightly from estimates from raw NB data (Fig. 1), exhibiting all the same qualitative patterns and slightly worse bias and precision. When *p_u_* = 0.5 (Fig. 2b), results exhibited similar, but more extreme, differences from the raw NB results.

### 3.3 Negative binomial data with under-reporting of zeroes

**Figure 3 pone-0000180-g003:**
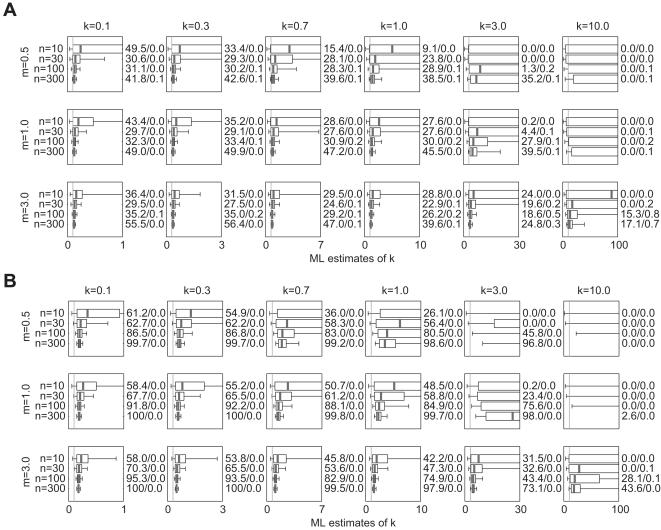
Estimated values of *k̂* and confidence interval coverage for NB datasets with under-reporting of zeroes. Individuals that caused no secondary infections were missed by surveillance with probability (a) *p_z_* = 0.2 and (b) *p_z_* = 0.5. 10,000 datasets were simulated as described in Section 2.1.3 of the text. Plotting details are described in Figure 1.

Results of estimation from NB surveillance datasets with under-reporting of the zero class, in which individuals who caused *x_i_* = 0 cases were omitted from simulated datasets with probability *p_z_*, are shown in Figure 3. For both *p_z_* = 0.2 (Fig. 3a) and *p_z_* = 0.5 (Fig. 3b), estimates of *k̂* are biased upward significantly. Notably, this effect does not diminish as sample size increases. Indeed, for most parameter sets the proportion of CI overestimates increases with higher *n*, as the sampling distribution narrows around the biased value.

### 3.4 Outbreak data

**Figure 4:**
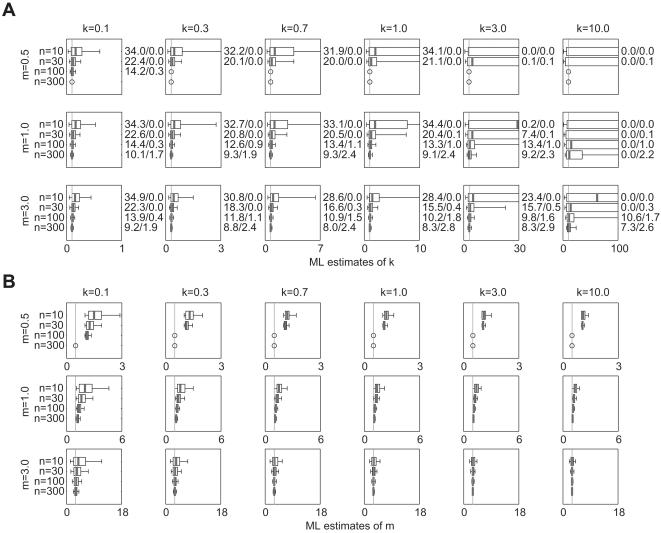
Estimated values of (a) *k̂* and (b) *m̂* for outbreak datasets generated by branching process simulations with NB offspring distributions. 10,000 datasets were simulated as described in Section 2.1.4 of the text. Circles indicate parameter sets for which fewer than 1 in 10^5^ simulated outbreaks had *n* cases or more. Other plotting details are described in Figure 1.

Estimates from simulated outbreak datasets are shown in Figure 4. For *m* = 0.5 and *k*>0.1, no results are presented for *n*≥100 because fewer than 1 in 10^5^ simulated outbreaks reached 100 cases. For other values of *m* and *k*, estimates of *k̂* are quite robust (Fig. 4a). Comparing these results to estimates from Figure 1, it is evident that estimates from outbreak datasets have similar biases (slightly positive for small *n*, but diminishing as *n* increases) and precisions that are as good and sometimes better than those from unaltered NB data. The outbreak datasets yield slightly more CI overestimates for *m* = 3, even though the IQR and [5^th^, 95^th^] percentile interval of the sampling distribution is often smaller. Outbreak datasets yield fewer CI overestimates for *k* = 0.1, *m* = 0.5 or 1.0, and *n* = 10 or 30.

ML estimates of the mean are shown for these datasets as well (Fig. 4b). There is a striking positive bias evident in the estimates of *m̂* for *m* = 0.5; in all cases shown, the distribution of *m̂* estimates has median value >1 and 5^th^ percentile value ≥1. For *m* = 1, there is an upward bias in the *m̂* estimates that decreases as sample size rises. For *m* = 3, the upward bias persists but is very slight for *k*≥0.3 or *n*≥30.

## Discussion

This study makes three novel contributions to the established literature on estimation of the NB dispersion parameter *k*. It provides the first comprehensive evaluation of ML estimation of *k* for highly overdispersed datasets (i.e. those with *k*<1); it reports the coverage accuracy of CIs derived from those estimates; and it examines potential biases in estimation due to methods and errors of data collection, with application to epidemiological datasets in particular and biological datasets in general. The major qualitative results are summarized in Table 1.

**Table 1:**
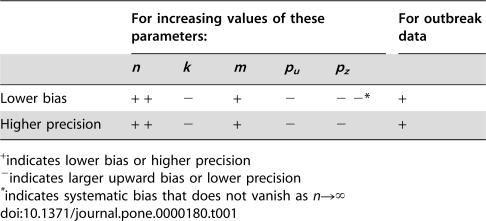
Influence of NB parameters and data types on bias and precision of *k̂*.

	For increasing values of these parameters:					For outbreak data
	*n*	*k*	*m*	*p_u_*	*p_z_*	
Lower bias	+ +	−	+	−	− −*	+
Higher precision	+ +	−	+	−	−	+

+ indicates lower bias or higher precision

− indicates larger upward bias or lower precision

* indicates systematic bias that does not vanish as *n*→∞

The results for unaltered NB datasets confirm and extend the findings of earlier studies. Small-sample estimates of *k̂* were biased toward overestimating *k*—and hence underestimating the degree of overdispersion in the data—as reported in previous studies using ML and related methods of estimation for *k*≥1 [14], [15], [17]. The positive bias in *k* arises because smaller samples are less likely to include values from the right-hand tail of the NB distribution, without which the dataset appears more homogeneous. Estimates of *k̂* were less biased and more precise for larger values of *m*, possibly because such datasets had higher total numbers of non-zero events. Estimates were more biased and less precise for higher values of *k* (particularly in the previously-studied range of *k*≥1), corresponding to the known instability of ML estimates when data are closer to being fitted by a Poisson distribution [13]. Intuitively, this effect arises because a NB distribution with *k* = 10 is qualitatively similar to one with *k* = 50 or *k*→∞, and quite dissimilar to one with *k* = 1, so the range of *k̂* estimates for small samples tends to be large and skewed upwards.

One previous simulation study [16] presented in-depth results for estimation of *k*<1 (specifically, for *k* = 0.4), employing method-of-moments estimates *k̂*_mom_ rather than the ML estimates assessed here. That study reported that smaller sample sizes from NB datasets led to systematic underestimation of the mean and variance and overestimation of *k*; the variance/mean ratio was also biased downward by small *n*. There is one interesting difference between the method-of-moments estimates results of Gregory and Woolhouse [16] and the present results for ML estimation: the positive bias of *k̂*_mom_ was fairly constant as *m* increased (though the range of *k̂*_mom_ values was greatest for lower *m*), while the bias of ML estimates *k̂* decreased for higher *m* (Fig. 1). It is notable that their values of *m* ranged from 1.25 to 160 (for *k* = 0.4), while the values used here ranged from 0.5 to 3 (for *k* between 0.1 and 10).

Several salient patterns emerged regarding the realized coverage of 90% CIs, as estimated using the asymptotic variance of ML estimates. The true coverage of the nominal 90% intervals was typically less than 90%, and CI overestimates were much more numerous than CI underestimates. For all parameter sets considered, <5% of CIs had upper bounds below the true value of *k*. The realized coverage of the CIs is driven by the interplay of two factors: the value of the estimates, *k̂*, and the breadth of the intervals (determined by the sampling variance, 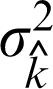). The upward bias of *k̂* increases for lower values of *n* and *m* and higher values of *k*; lower values of *n*, *m*, or *k* lead to increases in 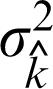 and hence broader intervals. Overestimates of *k̂* favor CI overestimates by setting a high mid-point for the estimated intervals, and by reducing the estimated sampling variance (because 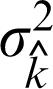 is calculated with an inflated value of *k*) and thus leading to narrower intervals. The gross patterns in the frequency of CI overestimates thus are driven primarily by patterns of bias in *k̂*.

To understand the finer patterns in CI coverage accuracy, particularly for CI underestimates and for CI overestimates for higher values of *k*, it is necessary to consider how the CIs are calculated. Recall that intervals were estimated for α = 1/*k* as [α̂−*z*_0.95_σ_α̂_, α̂+*z*_0.95_σ_α̂_], then converted into intervals for *k*. CI underestimates for *k* occur when α<α̂−*z*_0.95_σ_α̂_. The complete absence of CI underestimates in many small-*n* parameter sets arises because α̂<*z*_0.95_σ_α̂_ such that the lower bound of the CI for α̂ is <0. In these instances, the upper bound of the CI for *k̂* is set to the maximum value for *k̂* and cannot be exceeded. As *n*, *m*, or *k* increases, σ_α̂_ decreases and the CIs narrow such that some CI underestimates occur. Similarly, CI overestimates occur when α>α̂+*z*_0.95_σ_α̂_. As *k* increases, CI overestimates become less frequent (despite the high frequency of *k̂* overestimates) because α = 1/*k* is often smaller than *z*_0.95_σ_α̂_. Because α̂ is constrained to positive values in these simulations, CI overestimates are impossible when α<*z*_0.95_σ_α̂_. Accordingly, for given values of *k*>1, CI overestimates are more frequent for higher values of *n* and *m* (corresponding to lower values of σ_α̂_). This study's focus on overdispersed datasets, and hence on the positive values of *k* familiar to biologists, has thus influenced the determination of CI coverage in some regions of parameter space. Estimation procedures allowing for underdispersed data (α̂<0) may show different results. Investigators requiring CIs guaranteed to reach nominal levels of coverage should consult the literature on exact CIs for discrete distributions [e.g. 26].

The simulation results from surveillance and outbreak datasets (Figs. 2–4) can be interpreted readily in light of the raw NB results discussed above. For datasets where individual values correspond to completely unconnected events (e.g. epidemiological surveillance of multiple independent introductions of a disease, or many other biological observations), the effects of two forms of under-reporting were assessed. In uniform under-counting, each instance of the quantity being counted (e.g. secondary cases, in the epidemiological context) can be overlooked with equal probability *p_u_*. The expected value of each datum *x_i_* in the raw dataset (drawn from an NB distribution with parameters *m* and *k*) is reduced to (1−*p_u_*) *x_i_*, and the resulting distribution is NB with parameters (1−*p_u_*) *m* and *k* (as argued under the topic of ‘population-wide control measures’ by Lloyd-Smith et al. [8]). Thus uniform under-counting does not introduce systematic bias to ML estimates of *k*, but does cause a slight increase in the small-sample bias and decrease in precision (Fig. 2) corresponding to the effect of a lower mean, as characterized for raw NB data (Fig. 1).

In contrast, the second class of under-reporting bias, in which *x_i_* = 0 events are omitted from datasets with probability *p_z_*, leads to systematic overestimation of *k* that does not vanish as *n* increases (Fig. 3). NB distributions with low *k* are characterized by large zero classes and long tails (giving rise to the large variance-to-mean ratios that define overdispersion). Decreasing the proportion of zeroes (hence replacing *x_i_* = 0 events by *x_i_*>0 events) leads to higher sample mean *m̂* and lower sample variance *ŝ* ^2^. As is readily seen from the method-of moments estimator *k̂*_mom_ = *m̂* ^2^/(*ŝ* ^2^−*m̂*) [10], this will bias estimates of *k* to higher values. Investigators should be vigilant for this class of under-reporting bias, and conduct estimation using a zero-modified NB distribution [27] if zero under-counting is suspected.

Outbreak datasets involve a mechanism of data generation that is particular to epidemiological (or demographic) processes. Earlier analyses have shown that when offspring distributions are highly overdispersed (e.g. NB with *k*<1), the outbreaks that succeed tend to be those with early superspreading events [8]. The present results show that this does not cause underestimation of *k* as had been feared; estimates of *k̂* from outbreak data (Fig. 4a) exhibited similar properties to those from raw NB data (Fig. 1). Indeed, outbreak estimates had slightly smaller bias and greater precision for smaller *n*, probably because the use of outbreak data (biased toward including high-*x_i_* events) counteracts the usual small-sample bias (which arises because small datasets often lack high-*x_i_* events). Therefore the selection bias inherent in outbreak datasets acts to offset somewhat the usual upward bias in estimates of *k̂*.

In sharp contrast, estimation of *m̂* from outbreak datasets (assessed by simulation because, unlike the surveillance cases, the potential bias cannot be computed directly) is strongly biased upward when *m* is below or near 1 (Fig. 4b). This is unsurprising because the minimum value of *m̂* for an outbreak with *n* cases is (*n*−1)/*n* (for an outbreak that dies out immediately following the *n* ^th^ case), while higher values are quite feasible. (Recall that *m̂* is estimated as the mean number of secondary cases generated by the first *n* cases in an outbreak, regardless of whether the outbreak continues beyond *n* cases. If the cumulative number of cases after the *r* ^th^ generation of transmission is *j*, then the mean value of *x_i_* for *i* = 1 to *j* is (*j*−1)/*j*. If the *n* ^th^ case then occurs in the (*r*+1)^th^ generation of transmission, then all infections caused by the final *n*−*j* individuals in the dataset (i.e. *x_i_* for *i* = *j*+1 to *n*) serve to inflate *m̂* above its minimum value of (*n*−1)/*n.*) The greatest bias in *m̂* occurs for low *k* and *n*, when large superspreading events in the final generation can have disproportionate effect on the sample mean. For *m* = 1.0, the bias decreases as *n* increases, probably because higher-*n* datasets involve more generations of disease transmission, so the ‘left-over’ cases of the final generation (i.e. the final *n*−*j* individuals in the example above) make a smaller proportional contribution. For *m* = 3.0, there is no substantial bias for any parameters (with a minor exception for *k* = 0.1 and *n* = 10).

The results presented here suggest several avenues for future work. This study has focused on ML estimation only, and it would be fruitful to extend the conclusions to other methods of estimating *k*, such as maximum quasi-likelihood [14], method-of-moments with small-sample correction [16], or bias-corrected ML [17]. Further studies on estimation of *m̂* will be interesting, particularly in the epidemiological context where the mean of the offspring distribution is equivalent to the crucial quantity *R*_0_ [8], [22]. In particular, it will be important to learn how the overdispersion observed in disease transmission data [8] influences estimation of *R*_0_ from continuous-time outbreak data such as daily case reports [28], [29], as opposed to estimation directly from known chains of transmission as assessed here. Overdispersed offspring distributions cause outbreaks to either die out stochastically or grow explosively [8], so estimation of *R*_0_ from daily case reports (of successful outbreaks only, necessarily) may exhibit bias beyond that shown in Figure 4b.

In summary, this study showed that there is minimal risk of underestimating *k*—and hence of overestimating the degree of overdispersion in the data—due to small sample size or any of the three process biases considered here. There is substantial risk of overestimating *k*, particularly when sample sizes are small or the zero-class is systematically under-counted. All of the systematic biases identified in this study favored higher values of *k̂*, and instances when confidence intervals excluded the true value *k* were predominantly overestimates. Note that an independent risk of underestimating *k* can arise from pooling data from heterogeneous groups: the dispersion parameter estimated from pooled data is nearly always less than the average of values estimated for the individual groups [11], [16]. Regarding sample sizes for NB datasets with *k*≤1, *n* = 100 or more allows accurate and precise ML estimation of *k̂*, while for *n* = 30 the median estimates showed minimal bias but the sampling distribution skewed to high values. A sample size of 10 yields unreliable estimates, particularly for *m*≤1. These findings will help guide prospective design of sampling regimens, or, when sample size cannot be increased, will aid investigators in understanding the limitations of ML estimates of *k̂* and associated CIs.
